# A Qualitative Exploration of Hong Kong Medical Educators’ Perspectives on Factors Influencing Their Resilience

**DOI:** 10.5334/pme.1616

**Published:** 2025-09-24

**Authors:** Linda Chan, Paul Po Ling Chan, Fraide A. Ganotice, Julie Yun Chen, Tai Pong Lam, Carmen Ka Man Wong, Emma Victoria Marianne Bilney, Zoe Ho Wai Tang, Sam Cheuk Hong Yuen, Samuel Yeung Shan Wong, Cynthia R. Whitehead, George L. Tipoe

**Affiliations:** 1 Department of Family Medicine and Primary Care, Li Ka Shing Faculty of Medicine, The University of Hong Kong, Hong Kong, China; 2 The Bau Institute of Medical and Health Sciences Education, Li Ka Shing Faculty of Medicine, The University of Hong Kong, Hong Kong, China; 3 Department of Family Medicine and Primary Care, The University of Hong Kong – Shenzhen Hospital, Shenzhen, Guangdong, China; 4 The Jockey Club School of Public Health and Primary Care, The Chinese University of Hong Kong, Hong Kong, China; 5 The Wilson Centre for Research in Education, University Health Network and Temerty Faculty of Medicine, University of Toronto, Toronto, Canada; 6 Department of Family Medicine and Community Medicine, Temerty Faculty of Medicine, University of Toronto, Toronto, Canada; 7 Women’s College Hospital, Department of Family and Community Medicine, University of Toronto, Toronto, Canada

## Abstract

**Introduction:**

Globally, alarming trends of psychological distress among physicians and medical students threaten patient care and professionalism. The resilience and well-being of medical educators have been recognised as key influences on learners. However, relevant research is limited, especially in Asian contexts. Using the National Academy of Medicine (NAM) model as a lens, this study explores what external and individual factors impact the resilience of Hong Kong (HK)-based medical educators.

**Methods:**

HK-based medical educators, who taught medical students and physicians, were recruited using purposive sampling. They participated in semi-structured online interviews from 06/2021 to 04/2022. Anonymous sociodemographic information was collected through an online survey, and video recordings were transcribed anonymously. Guided by the NAM model, a hybrid deductive and inductive thematic analysis was conducted.

**Results:**

Twenty medical educators participated. They identified factors capturing all seven NAM model domains as influencing their resilience. Of those, “*organisational factors”* (institutional expectations, recognition, and rewards) and “*personal factors”* (social support from family, friends, and colleagues, and a sense of purpose in their roles) were perceived as influencing their resilience to a similar extent, suggesting that both organisational support and individual connections can bolster medical educators’ resilience.

**Discussion:**

This study, the first of its kind in Asia, examined the applicability and contextual suitability of the NAM model for use among HK-based medical educators. They perceived organisational and individual factors as complementary in influencing their resilience. Our findings highlighted the importance of considering both system- and individual-level aspects when designing strategies for promoting resilience in this population.

## Introduction

The resilience of medical educators is a global concern due to the high rates of professional burnout documented in extant literature [1,2,3,4,5]. Medical educators are tasked with multifaceted responsibilities, such as patient care, teaching, research, and administrative duties, often resulting in a sense of perpetual task overload [1,6,7]. They are additionally expected to maintain excellent academic performance and scholarly output to advance their careers [6], even at the expense of their own health and well-being. Consequently, many endure long working hours [4], emotional exhaustion [2], work-family conflict [8], and low job satisfaction [9], ultimately facing burnout [10]. Resilience can improve the ability to cope effectively with adversity and stress [11], serving as a protective factor and enabling medical educators to flourish in their careers.

### Resilience and Medical Educators

Resilience is commonly perceived as a dynamic trait in the healthcare professions [12]. At its core, resilience can be defined as the ability to bounce back from adversity [11], as well as the demonstration of adaptability, perseverance, and strength in the face of difficulties [13]. The challenges that medical educators face are multidimensional, stemming from the diverse array of responsibilities they shoulder. Moreover, continuously evolving healthcare systems and educational practices add complexity to their roles, requiring constant adaptation and innovation [14]. Educators often encounter heightened levels of stress [6], role ambiguity [15], and structural barriers [3], which can significantly impact their job satisfaction and overall well-being. By leveraging their resilience, medical educators can not only withstand the rigors of their profession but also foster creativity and maintain competency in medical education. However, while multiple studies have examined resilience among physicians [12,16,17] and medical students [18,19,20], limited research has focused on medical educators [1,4,8,21]. Furthermore, existing research in this field has been done in predominantly Western contexts.

Understanding resilience among medical educators necessitates a nuanced examination of the sociocultural factors that shape their experiences and responses to adversity [22,23]. Cultural norms, societal expectations, and organisational culture play significant roles in influencing the resilience of this group across different contexts and settings [24]. In the particular East Asian context of HK, cultural values such as collectivism, hierarchical structures, and relational harmony [25,26,27] may impact how medical educators perceive and manage stress, seek support, and prioritise self-care. Social norms that value emotional restraint and interdependence are reflected in the persistent stigma surrounding mental health [25,28,29]. Organisational cultures that promote open communication, collaboration, and support systems can enhance medical educators’ resilience and well-being [24]. Cultural sensitivity and inclusivity can help healthcare organisations remove unnecessary barriers which may otherwise undermine the resilience of educators [25,26]. A comprehensive framework to understand resilience among medical educators is therefore important.

### National Academy of Medicine (NAM) Model

The National Academic of Medicine (NAM) conceptual model is a comprehensive framework addressing the systemic- and individual-level factors associated with resilience and well-being among clinicians [30]. It encompasses both external and internal influences on clinician resilience and well-being across various healthcare professions and settings [31,32], supporting its selection as a useful tool to investigate the resilience of medical educators. Notably, the NAM model emphasises systemic factors within health systems and organisations, allocating five domains to external factors (*Healthcare Responsibilities; Learning/Practice Environment; Organisational Factors; Rules and Regulations; Society and Culture*) and two to internal factors (*Personal Factors; Skills and Abilities*). Using the NAM model as a lens can help us to examine the influences of these external and internal factors on medical educators’ resilience, particularly in different socio-cultural contexts.

The existing literature predominantly explores resilience among academics within a Western context [1,2,3,4,5,6,7,8,9,10,33], sidestepping the necessary socio-cultural considerations that may emerge in different national frameworks or geographical regions. The socio-cultural context in Asia, and specifically East Asia, is different from that in Western countries. Factors such as family expectations, social norms and workplace dynamics can uniquely affect the resilience of health professionals in this region [24,34], and a fuller understanding of these factors has significant implications for the international medical education community. Moreover, the findings from our study may offer insights to medical educators and their organisations in other East Asian countries (e.g., Japan, Korea), which share similar cultural outlooks on academic productivity [35,36].

By employing the NAM model as a lens, this study explores how systemic- and individual-level factors impact medical educators’ resilience. Furthermore, we attempt to illuminate the effect of culturally specific factors affecting resilience. Drawing attention to some prevalent cultural paradigms found in the East Asian context of HK may help to add extra dimensions to the existing discourse inspired by research in Western settings. With increasing awareness of diversity in academic medicine, medical educators often collaborate in multicultural workplaces. Research in an East Asian context can help to illuminate the perspectives that East Asian educators may bring with them into multicultural communities of practice elsewhere. Such insights are crucial for designing multifaceted faculty development programmes to support the resilience and well-being of medical educators in culturally nuanced ways. Therefore, our study aims to explore the impact of systemic and individual factors on medical educator resilience in an East Asian and specifically Hong Kong (HK) context. Through the lens of the NAM model [30], we explore what factors HK-based medical educators perceive as influencing their resilience.

## Methods

### Study Design

This study is the qualitative segment of a larger multi-phased, mixed-methods research project examining resilience among HK medical educators. Using purposive sampling, medical educators from HK’s two medical schools were recruited from June 2021 to April 2022. In general, these educators were predominantly Chinese, practising medicine, working in academic or honorary teaching positions, and otherwise had varied sociodemographic characteristics such as their age range and years of experience as medical educators. Specifically those who were engaged in teaching medical students, and/or physicians, were invited via email to attend online semi-structured individual interviews. Written informed consent was obtained and participants’ anonymity was guaranteed. Our study was conducted according to the Declaration of Helsinki. It was approved by the University of Hong Kong’s Human Research Ethics Committee (ref: EA200136) and the Joint Chinese University of Hong Kong-New Territories East Cluster Clinical Research Ethics Committee (ref: 2021.079). We also adhered to the consolidated criteria for reporting qualitative research [37] (COREQ; see Supplementary Appendix A).

### Data Collection

The interview guide (Supplementary Appendix B) was designed by the principal investigator (L.C.), a board-certified female family physician with an additional master’s in medical education, to delve into the perceived factors influencing the resilience of HK medical educators. All interviews were conducted in English by L.C. and video-recorded via Zoom (Zoom Video Communications, Inc., San Jose, California), and averaged 25 minutes each. Afterward, all recordings underwent transcription and anonymisation. Participant recruitment ended when the data gathered sufficiently addressed our research question. Our multidisciplinary team consisted of researchers from the biomedical and health sciences, clinical psychology, educational psychology, medicine, and medical education. This diversity of professional expertise, combined with the varied cultural perspectives of our team members, enriched the analytical and interpretative process.

### Data Analysis

A hybrid approach [38] of deductive analysis guided by the NAM model [30] and inductive analysis to explore the unique perspectives of our HK participants was used. Adopting Braun and Clarke’s approach [39], the research team members familiarised themselves with all transcripts by reading them twice. The lead research assistant (P.P.L.C.) then coded five transcripts using the NAM model and created an initial codebook that was tailored to the HK medical educator context (Supplementary Table 1). Subsequently, the research team of four collectively reviewed code definitions as well as the distinctions between codes and engaged in deliberations to refine the codebook. Each transcript was coded by two research team members to ensure agreement and consistency. Throughout this process, any discrepancies were addressed and resolved collaboratively. NVivo (Windows 14.23.2, Lumivero, Denver, Colorado) facilitated the qualitative analysis.

## Results

Table 1 summarises the sociodemographic characteristics of the 20 medical educators who participated. There was a balanced distribution in gender, with 10 (50%) male and female participants each. Eleven (55%) participants held a doctorate as their highest professional qualification, while the majority (*n*=12, 60%) were between 30 and 49 years old. Additionally, 17 participants (85%) identified as ethnically Chinese, were married, and worked full-time. Furthermore, 18 participants (90%) had 5–9 years of experience as medical educators.

**Table 1:** Characteristics of Participants.


PARTICIPANT CHARACTERISTICS (*N* = 20)	FREQUENCY (%)

**Age (in years)**	

20–29	–

30–39	6 (30%)

40–49	6 (30%)

50–59	5 (25%)

≥60	3 (15%)

**Gender**	

Male	10 (50%)

Female	10 (50%)

**Professional Background**	

Biomedical Sciences	2 (10%)

Medicine	10 (50%)

Nursing	3 (15%)

Pharmacy	3 (15%)

Other	2 (10%)

**Ethnicity**	

Chinese	17 (85%)

Caucasian	2 (10%)

Others	1 (5%)

**Relationship Status**	

Single	2 (10%)

Partnered	1 (5%)

Married	17 (85%)

**Highest Professional Qualification**	

Bachelor’s degree	–

Specialist qualification	1 (5%)

Master’s degree	7 (35%)

Doctorate	11 (55%)

Other	1 (5%)

**Years Worked as Health Professions Educator**	

0–4	2 (10%)

5–9	6 (30%)

10–14	4 (20%)

15–19	4 (20%)

≥ 20	4 (20%)

**Highest Qualification attained in Medical Education**	

None	7 (35%)

Certificate	3 (15%)

Diploma	–

Bachelor’s	–

Master’s or above	9 (45%)

Other	1 (5%)

**Employment Status**	

Full-time	17 (85%)

Part-time	–

Honorary	3 (15%)


Among the seven domains outlined in the NAM model, *Organisational Factors* arose as the primary external influence affecting medical educator resilience, followed closely by *Healthcare Responsibilities* and the *Learning/Practice Environment*. Of the two internal domains, *Personal Factors* appeared to exert the most significant impact. A finalised codebook and a list of representative quotes are presented in Table 2.

**Table 2:** Detailed Codebook: National Academy of Medicine (NAM) Model and Factors Influencing Hong Kong Medical Educators’ Resilience.


NAM DOMAINS	EXEMPLAR QUOTES

**Organisational Factors** This domain includes organisational structure/culture, and institutional expectations. It not only highlights the importance of institutional recognition and incentives but also the provision of employee support in medical educator resilience and wellness.	**[Q1]** “*if we have a good management team and the team is strong…to build up their resilience in the sense that they know they are caring for them*” – Participant 11 **[Q2]** “*if they are not recognised, they will be demoralised. And they absolutely lost the sense of direction…”* – Participant 13 **[Q3]** “*I am staying up late to finish something. I think it’s something that we [are] expected to achieve.”* – Participant 17

**Healthcare Responsibilities** This domain highlights medical educators’ responsibilities related to teaching, research, students, and administration.	**[Q4]** “*…Hong Kong medical educators [are] probably not fortunate enough to only take on a teaching role. So there’s a lot of administrative things to do…a lot of grant[s] to get and other things…*” – Participant 18

**Learning/Practice Environment** This domain includes workplace factors (e.g., team dynamics, workload, safety), and whether the practice environment is interdisciplinary.	**[Q5]** “*when there’s a conflict in the work situation, it becomes an enormous source of stress and can be very hindering and a major barrier*” – Participant 1 **[Q6]** “*…lower student-teacher ratio…very exhausting after consecutively teaching for 5 hours*” – Participant 3 **[Q7]** “*I think resilience is you’ll be [given] enough support…to create a right kind of teaching environment…*” – Participant 13

**Society & Culture** This domain includes sociocultural expectations in the Hong Kong context.	**[Q8]** “*…more like a Western concept to me…working in Hong Kong is actually stressful because of the.. working environment as well*” – Participant 10 **[Q9]** “*Asian culture, people are expected to be tough…work-life balance is very, very challenging and not well-addressed*” – Participant 17 **[Q10]** “*we tend to hold it to ourselves, if we discuss…maybe we will be seen as a bit incompetent or inadequate*.” – Participant 5

**Rules & Regulations** This domain includes concerns about health systems.	**[Q11]** “*…the existing health system [has] some handicap…*” – Participant 15

**Personal Factors** This domain includes medical educators’ sense of meaning and purpose in their roles, as well as interpersonal relationships and social support networks.	**[Q12]** “*I think talking with colleagues, and [seeking] support from friends are also very important, I think, social support is also one of the important factors [that] contributes to a higher resilience level*” – Participant 10 **[Q13]** “*it’s [a] difficult situation, especially [if] it’s family members [who] are having some problems*” – Participant 20 **[Q14]** “*So when we’re physically unwell. If we had pain or we’re not able to sleep, um…physically these things will affect our well-being a lot, and particularly if they [are] prolonged over time, it wears [us] out mentally, emotionally, spiritually*”– Participant 1

**Skills & Abilities** This domain includes coping strategies, self-imposed expectation management, and abilities to navigate new skills.	**[Q15]** “*…I think mindfulness is very helpful for teachers to build up the resilience and to live in the moment…*” – Participant 10 **[Q16]** “*I mean life has to go on. Medical education has to go on…we first need to manage our expectation…*” – Participant 5 **[Q17]** “…*to improve that resilience [we] should [develop] technical skills for and adopt IT and e-learning techniques.*.” – Participant 20


*Abbreviation*. NAM = National Academy of Medicine.

### Organisational Factors

Across all seven domains, organisational factors were most frequently referred to. Key themes within this domain underscored the importance of a robust management team characterised by strong, supportive leadership (Quote [Q]1):

*“if we have a good management team and the team is strong…to build up their resilience in the sense that they know they are caring for them”* (Participant [P]11)

Streamlining administrative procedures and eliminating bureaucratic obstacles were highlighted as essential for reducing stress and enhancing efficiency. Moreover, participants emphasised the need for increased incentives and recognition to bolster motivation, job satisfaction [Q2], and ultimately employee retention. They highlighted the necessity of clear and transparent career pathways to provide guidance and support professional development. Furthermore, participants expressed concerns about professional burnout, describing feelings of exhaustion and a lack of purpose, exacerbated by unwritten institutional-level expectations that employees would ‘go the extra mile’ without questioning [Q3]:

*“I am staying up late to finish something. I think it’s something that we [are] expected to achieve.”* (P17)

This underscored the need for the organisation to provide support for their members’ resilience and well-being.

### Healthcare Responsibilities

Participants highlighted the multitude of responsibilities encompassing teaching, research, student engagement, and administrative duties [Q4]:

*“…Hong Kong medical educators [are] probably not fortunate enough to only take on a teaching role. So there’s a lot of administrative things to do…a lot of grant[s] to get and other things…”* (P18)

Of particular significance was the teacher-student dyad, revealing the intricate relationship between educators and students. This dynamic was perceived not only to shape participants’ sense of responsibility as medical educators, but also played a pivotal role in building their professional identities. While professional identity could be a source of resilience, it was potentially a double-edged sword as it could predispose participants to over-commitment, a factor undermining resilience.

### Learning/Practice Environment

Participants emphasised several key elements within this domain. Firstly, workload, staffing, and team dynamics were identified as significant determinants affecting resilience [Q5]:

*“when there’s a conflict in the work situation, it becomes an enormous source of stress and can be very hindering and a major barrier”* (P1)

Additionally, the provision of debriefing sessions at work emerged as crucial for addressing stressors and fostering resilience. Further essential aspects were curriculum design, scheduling, and staff-student ratio, all of which played vital roles in shaping the educational experience and facilitating workload management for medical educators [Q6]:

*“…lower student-teacher ratio…very exhausting after consecutively teaching for 5 hours”* (P3)

Safety and hygiene protocols related to COVID-19 were also raised as essential considerations within the practice environment, directly impacting the resilience and well-being of clinician educators. Overall, participants regarded the presence of a supportive and well-resourced teaching environment as important in enhancing resilience [Q7]:

*“I think resilience is you’ll be [given] enough support…to create a right kind of teaching environment…”* (P13)

These insights underscored the multifaceted nature of the learning and practice environment and its impact on medical educator resilience.

### Society and Culture

Within this domain, resilience was perceived as a concept more commonly associated with Western cultures [Q8], suggesting potential disparities in understanding and application across different cultural contexts. In the context of HK, the living and working environments were described as particularly stressful due to factors such as the unspoken workplace expectations:

*“…working in Hong Kong is actually stressful because of the… working environment as well”* (P10)

Moreover, participants noted cultural expectations in East Asian societies, where individuals were often encouraged to exhibit “*toughness and resilience*” in the face of adversity, and perceived that this tended to exacerbate a lack of work-life balance for medical educators [Q9]:

*“Asian culture, people are expected to be tough…work-life balance is very, very challenging and not well-addressed”* (P17)

This cultural expectation that East Asians should be able to take on and resolve troubles and stresses on their own may contribute to reluctance among medical educators to openly discuss psychological struggles, as such discussions could be perceived as signs of incompetence [Q10]:

*“we tend to hold it to ourselves, if we discuss…maybe we will be seen as a bit incompetent or inadequate.”* (P5)

These insights underscored the critical role of societal and cultural factors in shaping medical educators’ resilience, highlighting the need for culturally sensitive approaches to supporting resilience in diverse contexts.

### Rules and Regulations

This domain was the least mentioned and participants expressed concerns about health policy decisions plus the broader health system [Q11]:

*“…the existing health system [has] some handicap…”* (P15)

Participants’ comments collectively highlighted the need for improvement of policies within the public health and healthcare systems e.g., community empowerment, resource allocation and the need for empathy in medical education towards vulnerable groups, to better support the resilience of all healthcare professionals including educators.

### Personal Factors

This domain was also frequently mentioned by participants. They identified a sense of meaning and purpose in their roles as crucial, which resonated with their sense of responsibility towards their work and students. Relationships and social support also emerged as a prominent theme, highlighting the significance of support from family members, friends, and colleagues in navigating challenges and boosting mental flexibility [Q12]:

*“I think talking with colleagues, and [seeking] support from friends are also very important, I think, social support is also one of the important factors [that] contributes to a higher resilience level”* (P10)

While acknowledging the benefits of supportive family environments, participants also mentioned that complex family dynamics could pose challenges to resilience, highlighting the dual nature of family relationships as sources of both support and stress [Q13]:

*“it’s [a] difficult situation, especially [if] it’s family members [who] are having some problems”* (P20)

Furthermore, participants discussed the importance of physical, mental, and spiritual well-being in resilience building efforts. For example, challenges to physical health were noted to detract from overall resilience [Q14]:

*“So when we’re physically unwell. If we had pain or we’re not able to sleep, um…physically these things will affect our well-being a lot, and particularly if they [are] prolonged over time, it wears [us] mentally, emotionally, spiritually”* (P1)

These insights emphasised the importance of holistic approaches in supporting the resilience of medical educators.

### Skills and Abilities

For this domain, participants articulated that coping strategies (e.g., exercise, mindfulness, effective time- and stress-management) were essential tools for building resilience in the event of adversity [Q15]:

*“…I think mindfulness is very helpful for teachers to build up the resilience and to live in the moment…”* (P10)

The capacity to move on and overcome obstacles was also mentioned, reflecting a resilient mentality [Q16]:

*“I mean life has to go on. Medical education has to go on…we first need to manage our expectation…”* (P5)

Interestingly, self-imposed expectation management was recognised as a critical component in building resilience, with participants highlighting the importance of setting realistic goals and boundaries to mitigate stress and enhance resilience. Another specific example of a skill supportive of resilience emerged in terms of the ability to master new technologies, particularly in the context of the COVID-19 pandemic. Participants recognised the importance of adapting to e-learning techniques to navigate challenges posed by remote teaching [Q17]:

*“…to improve that resilience [we] should [develop] technical skills for and adopt IT and e-learning techniques…”* (P20)

## Discussion

To our knowledge, this is the first study in Asia and specifically in HK, that explored the factors perceived by medical educators as influencing their resilience. Their insights generally mapped well to the seven domains outlined in the NAM model [30], which considers systemic- and individual-level factors contributing to medical educator resilience. Our study enriches the current Euro-American perspective [40] by providing empirical evidence from an East Asian context, illustrating that both organisational and personal factors play equally significant roles in shaping medical educators’ resilience. In a collectivist context like HK, these organisational and interpersonal connections may be even more salient, as individuals are often embedded within dense social networks that shape their sense of purpose, responsibility, and well-being. The assumption of mutual support and shared accountability may make social and systemic resilience supports feel more intuitive or expected than in more individualist cultures. Conversely, social norms relating to the sharing of knowledge and the nature of interpersonal trust, as well as the high pressure to appear competent in all circumstances in an East Asian setting may at times become an additional drain on resilience compared with what may be expected in a Western context. By highlighting these culturally-embedded dynamics, our findings contribute to a more nuanced understanding of resilience that augments existing Western-centric models derived from more individualist contexts. In particular, the collectivist ethos of HK’s cultural context suggests that relational and organisational supports may be more deeply woven into the everyday experience of educators, sometimes reinforcing resilience through connection, belonging, and mutual responsibility, but at other times undermining it through excessive conforming pressure to appear competent.

In light of increasing globalisation of academic medicine, marked by the migration of health professionals and cross-border faculty appointments, our findings may benefit international efforts to better understand how culturally embedded organisational and personal factors interact to shape educator resilience. Specifically, both organisational structures and interpersonal relationships are central to resilience and acknowledging both commonalities, as well as context-specific dynamics can inform more inclusive and adaptable resilience-support strategies across diverse academic settings.

### Systemic Level

Among five external factors in the NAM model [30], the *Organisational Factors* domain emerged as paramount, with participants highlighting the significance of effective institutional management characterised by strong, supportive leadership and streamlined administrative processes. This finding resonates with existing literature, which has reported that only 3% of academic physicians found administrative duties meaningful, and prolonged hours spent on such tasks can predispose them to stress and burnout [6]. Similarly, Rao’s study [7] revealed a positive relationship between administrative workload and job dissatisfaction. Moreover, the call for a clear career track and recognition underscores participants’ narratives about feeling undervalued and disoriented in their professional development. Despite expectations for medical educators to excel in clinical practice, research, and teaching, assuming that their teaching duties will not impact their research output is unrealistic [34]. This echoes what participants addressed in the *Healthcare Responsibilities* domain, where a diverse array of responsibilities encompassing teaching, research, and administrative duties reflected the complexity of their roles and the need for comprehensive support strategies in fostering their resilience.

The competitiveness inherent in academic medicine, demanding excellence in clinical performance and teaching activities [1,2,3,4,5,10,35,36,41], alongside a prolific output of scholarly publications, may undermine resilience among medical educators. This aligns with findings identified from the *Learning/Practice Environment* domain, highlighting the impact of workload, staffing, and team dynamics on the resilience of HK-based medical educators. This phenomenon can be understood through the lens of socio-cognitive career theory (SCCT) [42,43], which suggests that contextual factors, like environmental features, shape career aspirations. Studies by Bakken et al. [44] and O’Sullivan et al. [45] applied SCCT in the context of academic medicine and revealed that conflicting demands plus expectations, insufficient early career support, and poorly signposted career pathways were detrimental to the job fulfilment of medical educators. These challenges could lead to diminished self-efficacy and over-commitment [34], thereby undermining resilience.

Nonetheless, the emphasis placed on the teacher-student relationship within the *Healthcare Responsibilities* domain, as collectively mentioned by participants, underscores its role in shaping medical educators’ sense of responsibility and professional identity [15]. Participants reported that their responsibilities towards students and the organisation helped them become resilient and fulfilled [9]. It can be argued that the intrinsic value of medical education could outweigh the drawbacks associated with overwhelming institutional demands [3,9,34]. However, academic medicine still predominantly places emphasis on research productivity, where research excellence often takes precedence over teaching excellence. Therefore, structured career pathways are deemed essential for guiding professional development, enhancing job satisfaction, and mitigating feelings of burnout and purposelessness caused by institutional expectations [9].

The *Society and Culture* domain revealed that individuals may face additional stress when they are expected to demonstrate resilience in order to cope with the demands of fast-paced living and working environments. This mindset is heavily influenced by the emphasis on interdependence in Chinese culture, where individuals feel obligated to maintain social cohesion [46]. Consequently, they may feel compelled to appear resilient, striving to meet unrealistic expectations to avoid disturbing social harmony [46]. This pressure to align with societal expectations places additional strain on medical educators, increasing their vulnerability to psychological distress [47]. In line with SCCT, culture acts as a contextual barrier, influencing how medical educators navigate the challenges [43] that impact their resilience [34]. The cultural expectations of toughness and resilience contribute to their reluctance to openly discuss psychological struggles, underscoring the importance of culturally sensitive approaches to supporting resilience. The *Rules and Regulations* domain was mentioned least of all, but comments made in this category reflected concerns about the limitations of the health system and the need for reform to better address the needs of patients and communities. Overall, these findings reflect the complex interplay of systemic factors that shape medical educator resilience.

### Individual Level

Corresponding to the socio-ecological resilience perspective [48], participants indicated the significance of supportive relationships in both professional and personal realms. This is consistent with prior literature highlighting the positive impact of social support on resilience outcomes [32], offering additional benefits in terms of performance and function. Although supportive family relationships enhance resilience, challenges like family responsibilities and conflicts can be detrimental, which echoes the findings of research linking work-family conflict to burnout and resilience [2,8,49]. Furthermore, prioritising mental, physical, and spiritual well-being emerged as crucial for resilience building. Participants acknowledged the advantages of activities like exercise and mindfulness, which is consistent with existing literature emphasising healthy lifestyles in reducing burnout and enhancing resilience [50].

Coping skills and resilience-building practices within the *Skills and Abilities* domain were identified as important. The interconnectedness between coping skills and resilience-building practices highlights their complementary nature, as seen in palliative medicine research, where self-awareness predicted physicians’ coping abilities and burnout propensity [51]. Furthermore, the overlap identified within and between these individual-level factors underscore their interconnectedness in bolstering resilience. For example, teamwork and communication skills, typically considered under *Skills and Abilities*, were also mentioned within the *Personal Factors* domain, reflecting their role in fostering social support and relationships.

### Limitations and Strengths

Our study is not without limitations. The sample size of 20 participants may limit the transferability of the findings to a broader population of medical educators in HK, as may the fact that the sample was confined to the academic medicine segment and did not generally cover either private practice or the public health sector. Participants included clinician-educators plus scientist-educators, and their scope of work could influence the issues raised. This may be a limitation but also a strength in that diverse views were represented. Moreover, our study did not consider gender differences, which have been shown to play a role in stress levels among academics with regards to research productivity, teaching, and clinical responsibilities [8,49]. Finally, although the NAM model was used as a conceptual framework to guide the analysis, this may also have inadvertently restricted our perceptions as researchers during the analysis process. Despite these limitations, our study offered valuable insights into the factors influencing HK medical educators’ resilience. Additionally, it demonstrated robust evidence that the NAM model [30] can serve as a comprehensive framework for understanding the facilitators and barriers to supporting resilience among this community of practice. Lastly, our study was not aimed at making broad cultural claims but sought to provide a foundation that encourages further research into medical educators’ well-being in diverse cultural contexts.

### Implications

The increasing trend towards globalised medical education workforces highlights the importance of diverse cultural understandings of resilience. This augments the current literature on resilience in academic medicine, traditionally viewed through a Western-focused lens. Our findings offer a valuable East Asian perspective that foregrounds the significance of both organisational and personal factors. In particular, the collectivist cultural context of HK suggests that resilience is naturally supported through strong relational ties, communal responsibility, and a sense of interconnectedness within institutions, but undermined by intense pressure to appear competent despite unrealistic expectations. These insights point to the need for resilience-support initiatives that account for both individual experiences and culturally embedded institutional structures, not only in this locality of East Asia but also in global academic environments shaped by the migration of health professionals. Furthermore, our study can potentially be replicated in other Asian territories that share similar workplace competitiveness as HK [35,36], aiding in mapping out the risk and protective factors in academic medicine that account for contextual or culturally specific considerations.

Given the multifaceted and dynamic nature of resilience, initiatives to promote medical educator resilience should be two-pronged – addressing personal factors, along with broader organisational and sociocultural influences. This is necessary because external factors may perpetuate issues in workplaces that are perceived to be dehumanising and unsupportive. Building a workplace environment that fosters a sense of purpose and connectedness requires collective action [52], with leaders and senior colleagues playing a key role in driving changes through wellness-centred leadership. This highlights the value of formal medical leadership development programmes that empower senior management to create supportive and inclusive workplaces that help medical educators to thrive. Implementing constructive changes also requires effort in addressing systemic negative issues, for example by promoting a culture of openness to discuss vulnerabilities in the workplace [47]. In parallel, medical educators may be encouraged to invest in their own self-care, monitor their own resilience, and be willing to act on warning signs of personal psychological distress [47].

## Conclusion

Our study underscores organisational and personal domains as being crucial in the support of resilience among medical educators. Furthermore, our findings spotlight the importance of broader and more multicultural understandings of factors that influence resilience, which can help cultivate more inclusive environments for fostering medical educator resilience in HK and beyond.

## Data Accessibility Statement

The dataset is available from the corresponding author upon reasonable request.

## Additional File

The additional file for this article can be found as follows:

10.5334/pme.1616.s1 Supplementary files. Appendix A, B and Table 1.

## Previous Presentations

Relevant parts of this research were previously presented on January 14, 2023 at the Tripartite Medical Education Conference in Hong Kong.

## References

[1] García-Rivera BR, Mendoza-Martínez IA, García-Alcaraz JL, et. al. Influence of resilience on burnout syndrome of faculty professors. Int J Environ Res Public Health. 2022;19(2):910. DOI: 10.3390/ijerph19020910 35055731 PMC8776145

[2] Porter M, Hagan H, Klassen R, et. al. Burnout and resiliency among family medicine program directors. Fam Med. 2018;50(2):106–112. DOI: 10.22454/FamMed.2018.836595 29432625

[3] Cora-Bramble D, Zhang K, Castillo-Page L. Minority faculty members’ resilience and academic productivity: are they related? Acad Med. 2010;85(9):1492–1498. DOI: 10.1097/ACM.0b013e3181df12a9 20453809

[4] McDermid F, Peters K, Daly J, Jackson D. Developing resilience: Stories from novice nurse academics. Nurse Educ. Today. 2016;38:29–35. DOI: 10.1016/j.nedt.2016.01.002 26860520

[5] Spilg EG, McNeill K, Sabri E, et. al. A cross-sectional study of the interrelationship between burnout, empathy and resilience in academic physicians. Psychol Health Med. 2022;27(8):1813–1820. DOI: 10.1080/13548506.2021.1954670 34281438

[6] Nassar AK, Reid S, Kahnamoui K, et. al. Burnout among academic clinicians as it correlates with workload and demographic variables. Behav. Sci. 2020;10(6):94. DOI: 10.3390/bs10060094 32471265 PMC7349515

[7] Rao SK, Kimball AB, Lehrhoff SR, et. al. The impact of administrative burden on academic physicians: results of a hospital-wide physician survey. Acad Med. 2017;92(2):237–243. DOI: 10.1097/ACM.0000000000001461 28121687

[8] Patel SI, Ghebre R, Dwivedi R, et. al. Academic clinician frontline-worker wellbeing and resilience during the COVID-19 pandemic experience: Were there gender differences? Prev Med Rep. 2023;36:102517. DOI: 10.1016/j.pmedr.2023.102517 38116283 PMC10728464

[9] Shanafelt TD, West CP, Sloan JA, et. al. Career fit and burnout among academic faculty. Arch Intern Med. 2009;169(10):990–995. DOI: 10.1001/archinternmed.2009.70 19468093

[10] Tijdink JK, Vergouwen AC, Smulders YM. Emotional exhaustion and burnout among medical professors; a nationwide survey. BMC Med Educ. 2014;14:1–7. DOI: 10.1186/1472-6920-14-183 25189761 PMC4167137

[11] Wu G, Feder A, Cohen H, et. al. Understanding resilience. Front. Behav. Neurosci. 2013;7:10. DOI: 10.3389/fnbeh.2013.00010 23422934 PMC3573269

[12] Cleary M, Kornhaber R, Thapa DK, West S, Visentin D. The effectiveness of interventions to improve resilience among health professionals: A systematic review. Nurse Educ. Today. 2018;71:247–263. DOI: 10.1016/j.nedt.2018.10.002 30342300

[13] Connor KM, Davidson JR. Development of a new resilience scale: The Connor-Davidson resilience scale (CD-RISC). Depress. Anxiety. 2003;18(2):76–82. DOI: 10.1002/da.10113 12964174

[14] Simpson D, Marcdante K, Morzinski J, et. al. Fifteen years of aligning faculty development with primary care clinician–educator roles and academic advancement at the Medical College of Wisconsin. Acad Med. 2006;81(11):945–953. DOI: 10.1097/01.ACM.0000242585.59705.da 17065852

[15] Lee SL, Rees CE, O’Brien BC, Palermo C. Identities and roles through clinician-educator transitions: A systematic narrative review. Nurse Educ. Today. 2022;118:105512. DOI: 10.1016/j.nedt.2022.105512 36054976

[16] McKinley N, Karayiannis PN, Convie L, et. al. Resilience in medical doctors: a systematic review. Postgrad Med J. 2019;95(1121):140–147. DOI: 10.1136/postgradmedj-2018-136135 30926716

[17] Fox S, Lydon S, Byrne D, et. al. A systematic review of interventions to foster physician resilience. Postgrad Med J. 2018;94(1109):162–170. DOI: 10.1136/postgradmedj-2017-135212 29018095

[18] Dunn LB, Iglewicz A, Moutier C. A conceptual model of medical student well-being: promoting resilience and preventing burnout. Acad Psychiatry. 2008;32:44–53. DOI: 10.1176/appi.ap.32.1.44 18270280

[19] Li ZS, Hasson F. Resilience, stress, and psychological well-being in nursing students: A systematic review. Nurse Educ. Today. 2020;90:104440. DOI: 10.1016/j.nedt.2020.104440 32353643

[20] Thompson G, McBride RB, Hosford CC, Halaas G. Resilience among medical students: the role of coping style and social support. Teach. Learn. Med. 2016;28(2):174–182. DOI: 10.1080/10401334.2016.1146611 27064719

[21] Nurse-Clarke N, Sockol LE. An Exploration of Resiliency Among Nurse Educators During the COVID-19 Pandemic. Nurs Educ Perspect. 2022 Sep 1;43(5):283–286. DOI: 10.1097/01.NEP.0000000000001014 35947139

[22] Ungar M. Cultural Dimensions of Resilience among Adults. In: Reich JW, Zautra AJ & Hall JS, editors. Handbook of Adult Resilience. New York: Guilford Press. 2010. pp. 404–423.

[23] Chan L, Dennis A. Resilience: a nationwide study of medical educators. MedEdPublish. 2019;8:20. DOI: 10.15694/mep.2019.000020.2 38089278 PMC10712508

[24] Clauss-Ehlers CS, Chiriboga DA, Hunter SJ, Roysircar G, Tummala-Narra P. APA Multicultural Guidelines executive summary: Ecological approach to context, identity, and intersectionality. Am Psychol. 2019;74(2):232–244. DOI: 10.1037/amp0000382 30762387

[25] Papadopoulos C, Foster J, Caldwell K. ‘Individualism-collectivism’ as an explanatory device for mental illness stigma. Community Ment. Health J. 2013;49:270–280. DOI: 10.1007/s10597-012-9534-x 22837106

[26] Kirmayer LJ, Sehdev M, Whitley R, Dandeneau SF, Isaac C. Community resilience: Models, metaphors and measures. Int J Indig Health. 2009;5(1):62–117. DOI: 10.18357/IJIH51200912330

[27] Kuo BC. Interdependent and relational tendencies among Asian clients: Infusing collectivistic strategies into counselling. Guidance and Counselling. 2004;19(4):158.

[28] Lei Y, Duan C, Shen K. Development and validation of the Chinese mental health value scale: a tool for culturally-informed psychological assessment. Front. Psychol. 2025;16:1417443. DOI: 10.3389/fpsyg.2025.1417443 40196204 PMC11973265

[29] Gao G. “Don’t take my word for it.”—understanding Chinese speaking practices. Int J Intercult Rel. 1998;22(2):163–86. DOI: 10.1016/S0147-1767(98)00003-0

[30] Brigham T, Barden C, Dopp AL, et. al. A journey to construct an all-encompassing conceptual model of factors affecting clinician well-being and resilience. NAM Perspect. 2018;8(1):201801b. DOI: 10.31478/201801b

[31] Stewart MT, Reed S, Reese J, Galligan MM, Mahan JD. Conceptual models for understanding physician burnout, professional fulfillment, and well-being. Curr Probl Pediatr Adolesc Health Care. 2019;49(11):100658. DOI: 10.1016/j.cppeds.2019.100658 31629639

[32] Munn LT, Huffman CS, Connor CD, et. al. A qualitative exploration of the National Academy of medicine model of well-being and resilience among healthcare workers during COVID-19. J Adv Nurs. 2022;78(8):2561–2574. DOI: 10.1111/jan.15215 35285054 PMC9111620

[33] Kumar K, Roberts C, Thistlethwaite J. Entering and navigating academic medicine: academic clinician-educators’ experiences. Med Educ. 2011;45(5):497–503. DOI: 10.1111/j.1365-2923.2010.03887.x 21486325

[34] Edwards MS, Ashkanasy NM. Emotions and failure in academic life: Normalising the experience and building resilience. J Manag Organ. 2018;24(2):167–188. DOI: 10.1017/jmo.2018.20

[35] Chatani Y, Nomura K, Horie S, et. al. Effects of gaps in priorities between ideal and real lives on psychological burnout among academic faculty members at a medical university in Japan: a cross-sectional study. Environ Health Prev Med. 2017;22(32):1–7. DOI: 10.1186/s12199-017-0626-7 29165115 PMC5664446

[36] Seo JH, Bae HO, Kim BJ, et. al. Burnout of faculty members of medical schools in Korea. J Korean Med Sci. 2022;37(9):e74. DOI: 10.3346/jkms.2022.37.e74 35257529 PMC8901883

[37] Tong A, Sainsbury P, Craig J. Consolidated criteria for reporting qualitative research (COREQ): a 32-item checklist for interviews and focus groups. Int J Qual Health Care. 2007;19(6):349–357. DOI: 10.1093/intqhc/mzm042 17872937

[38] Fereday J, Muir-Cochrane E. Demonstrating Rigor Using Thematic Analysis: A Hybrid Approach of Inductive and Deductive Coding and Theme Development. Int. J. Qual. Methods. 2006;5(1):80–92. DOI: 10.1177/160940690600500107

[39] Braun V, Clarke V. Using thematic analysis in psychology. Qual Res Psychol. 2006;3(2):77–101. DOI: 10.1191/1478088706qp063oa

[40] Wondimagegn D, Whitehead CR, Cartmill C, et. al. Faster, higher, stronger – together? A bibliometric analysis of author distribution in top medical education journals. BMJ Glob. Health. 2023;8(6):e011656. DOI: 10.1136/bmjgh-2022-011656 PMC10367082 37321659

[41] Serrano H, Andrea SJ, Lopes J, et. al. A qualitative investigation of burnout and well-being among faculty and residents in a Canadian psychiatry department. Acad Psychiatry. 2023;47(2):159–163. DOI: 10.1007/s40596-023-01745-1 36752998 PMC9907865

[42] Lent RW, Brown SD, Hackett G. Toward a unifying social cognitive theory of career and academic interest, choice, and performance. J. Vocat. Behav. 1994;45(1):79–122. DOI: 10.1006/jvbe.1994.1027

[43] Lent RW, Brown SD, Hackett G. Contextual supports and barriers to career choice: A social cognitive analysis. J. Couns. Psychol. 2000;47(1):36–49. DOI: 10.1037//0022-0167.47.1.36

[44] Bakken LL, Byars-Winston A, Wang MF. Viewing clinical research career development through the lens of social cognitive career theory. Adv Health Sci Educ Theory Pract. 2006;11:91–110. DOI: 10.1007/s10459-005-3138-y 16583288

[45] O’Sullivan PS, Niehaus B, Lockspeiser TM, Irby DM. Becoming an academic doctor: perceptions of scholarly careers. Med Educ. 2009;43(4):335–341. DOI: 10.1111/j.1365-2923.2008.03270.x 19335575

[46] Markus HR, Kitayama S. Culture and the Self: Implications for Cognition, Emotion, and Motivation. In: Altbach PG, Arnold K & King IC, editors. College Student Development and Academic Life: Psychological, Intellectual, Social and Moral Issues. New York: Routledge. 2014. pp. 264–293.

[47] LaDonna KA, Cowley L, Touchie C, LeBlanc VR, Spilg EG. Wrestling With the Invincibility Myth: Exploring Physicians’ Resistance to Wellness and Resilience-Building Interventions. Acad Med. 2022;97(3):436–443. DOI: 10.1097/ACM.0000000000004354 34380930

[48] Fletcher D, Sarkar M. Psychological resilience: A review and critique of definitions, concepts and theory. Eur. Psychol. 2013;18(1):12–23. DOI: 10.1027/1016-9040/a000124

[49] Carr PL, Ash AS, Friedman RH, et. al. Relation of family responsibilities and gender to the productivity and career satisfaction of medical faculty. Ann Intern Med. 1998;129(7):532–538. DOI: 10.7326/0003-4819-129-7-199810010-00004 9758572

[50] Beltman S, Poulton E. “Take a step back”: Teacher strategies for managing heightened emotions. Aust Educ Res. 2019;46:661–679. DOI: 10.1007/s13384-019-00339-x

[51] Sansó N, Galiana L, Oliver A, et. al. Palliative care professionals’ inner life: exploring the relationships among awareness, self-care, and compassion satisfaction and fatigue, burnout, and coping with death. J Pain Symptom Manage. 2015;50(2):200–207. DOI: 10.1016/j.jpainsymman.2015.02.013 25701688

[52] Winkel AF, Honart AW, Robinson A, Jones AA, Squires A. Thriving in scrubs: a qualitative study of resident resilience. Reprod. Health. 2018;15:1–8. DOI: 10.1186/s12978-018-0489-4 29587793 PMC5869777

