# Supplementary material for: A Qualitative Exploration of Hong Kong Medical Educators’ Perspectives on Factors Influencing Their Resilience
Source: Perspect Med Educ. 2025 Sep 24;14(1):590–602. doi: 10.5334/pme.1616 (PMC12466334; doi:10.5334/pme.1616)
Supplement: Supplementary files. — Appendix A, B and Table 1. [file pme-14-1-1616-s1.pdf]

Supplementary Appendix A. COREQ (Consolidated Criteria for Reporting Qualitative Research) Checklist

Topic	Item No	Guide Questions/Description	Page No
**Domain 1: Research team and reflexivity**			
Personal Characteristics			
Interviewer/facilitator	1	Which author/s conducted the interview or focus group?	8; Supplementary Appendix B
Credentials	2	What were the researcher's credentials? E.g., PhD, MD	8; Supplementary Appendix B
Occupation	3	What was their occupation at the time of the study?	8; Supplementary Appendix B
Gender	4	Was the researcher male or female?	8
Experience and training	5	What experience or training did the researcher have?	8; Supplementary Appendix B
Relationship with participants			
Relationship established	6	Was a relationship established prior to study commencement?	Supplementary Appendix B
Participant knowledge of the interviewer	7	What did the participants know about the researcher? e.g., personal goals, reasons for doing the research	Supplementary Appendix B
Interviewer characteristics	8	What characteristics were reported about the interviewer/facilitator? e.g., Bias, assumptions, reasons and interests in the research topic	Supplementary Appendix B
**Domain 2: Study design**			
Theoretical framework			
Methodological orientation and Theory	9	What methodological orientation was stated to underpin the study? e.g., grounded theory, discourse analysis, ethnography, phenomenology, content analysis	8-9
Participant selection			
Sampling	10	How were participants selected? e.g., purposive, convenience, consecutive, snowball	8
Method of approach	11	How were participants approached? e.g., face-to-face, telephone, mail, email	8
Sample size	12	How many participants were in the study?	9
Non-participation	13	How many participants refused to participate or dropped out? Reasons?	N/A
Setting			
Setting of data collection	14	Where was the data collected? e.g., home, clinic, workplace	8
Presence of non-participants	15	Was anyone else present besides the participants and researchers?	N/A
Description of sample	16	What are the important characteristics of the sample? E.g., demographic data, date	9; Table 1

Topic	Item No	Guide Questions/Description	Page No
Data collection			
Interview guide	17	Were questions, prompts, guides provided by the authors? Was it pilot tested?	8; Supplementary Appendix B
Repeat interviews	18	Were repeat interviews carried out? If yes, how many?	N/A
Audio/visual recording	19	Did the research use audio or visual recording to collect the data?	8
Field notes	20	Were field notes made during and/or after the interview or focus group?	N/A
Duration	21	What was the duration of the interviews or focus group?	8
Data saturation	22	Was data saturation discussed?	8
Transcripts returned	23	Were transcripts returned to participants for comment and/or correction?	N/A
**Domain 3: analysis and findings**			
Data analysis			
Number of data coders	24	How many data coders coded the data?	9
Description of the coding tree	25	Did authors provide a description of the coding tree?	Table 2; Supplementary Table 1
Derivation of themes	26	Were themes identified in advance or derived from the data?	9
Software	27	What software, if applicable, was used to manage the data?	9
Participant checking	28	Did participants provide feedback on the findings?	N/A
Reporting			
Quotations presented	29	Were participant quotations presented to illustrate the themes/findings? Was each quotation identified? e.g., participant number	10-15; Table 2; Supplementary Table 1
Data and findings consistent	30	Was there consistency between the data presented and the findings?	9-19
Clarity of major themes	31	Were major themes clearly presented in the findings?	9-15; Table 2
Clarity of minor themes	32	Is there a description of diverse cases or discussion of minor themes?	13; 18

Developed from: Tong A, Sainsbury P, Craig J. Consolidated criteria for reporting qualitative research (COREQ): a 32-item checklist for interviews and focus groups. *International Journal for Quality in Health Care*. 2007. Volume 19, Number 6: pp. 349 – 357

Supplementary Appendix B. Structured Interview Guide

The purpose of this semi-structured interview is to explore medical educators' conceptualisations of resilience; factors perceived as promoting and undermining their resilience; the processes by which this occurs; and the interventions they consider as beneficial for fostering resilience. The following are the set of tentative guide questions to be asked:

1) In the context of medical education, what does 'resilience' mean to you?
2) What factors or strategies help build your resilience?

2b) How/what are the processes by which the above factors or strategies help to build your resilience?

3) What factors, concerns, and/or circumstances undermine your resilience?

3b) How/what are the processes by which the above factors, concerns, and/or circumstances undermine your resilience?

4) Are there any interventions you consider as beneficial for fostering resilience?

Interviewer Characteristics and Reflexivity

The principal investigator (PI; L.C.) conducted all the semi-structured individual Zoom interviews. As an academic family physician with a master's in medical education, her research interests centre on medical educators' resilience and well-being. Participants understood L.C.'s rationale for the study and the researcher-participant relationship was established at each interview.

Supplementary Table 1. Initial Codebook: National Academy of Medicine (NAM) Model and Factors Influencing Hong Kong Medical Educators' Resilience

	NAM Domains	Examples of NAM Definitions	Application of NAM Definitions to Medical Educators' Resilience Data	Exemplar Quotes
**External Factors**	**Organisational Factors**	▪ Bureaucracy & power dynamics<br>▪ Culture, leadership, and staff engagement<br>▪ Level of support for healthcare team<br>▪ Professional development opportunities<br>▪ Workload, performance, compensation & value attributed to work elements	o Organisational structure/culture<br>o Institutional□ level expectations and standards<br>o Employee support related to well□ being/resilience<br>o Recognition & rewards	"*...we need appreciation, we need rewards for our efforts*." – Participant 6
	**Healthcare Responsibilities**	▪ Teaching & research opportunities<br>▪ Student/trainee responsibilities<br>▪ Clinical responsibilities<br>▪ Administrative responsibilities	o Responsibilities related to teaching, research, students and administration<br>o Teacher-student dyad<br>o Research funding opportunities	*"…not fortunate enough to only take on [a] teaching role…lots of administrative things to do*" – Participant 18
	**Learning/Practice Environment**	▪ Team structure & functionality<br>▪ Collaborative vs. Competitive<br>▪ Learning & practice setting<br>▪ Curriculum, Mentorship<br>▪ Workplace safety	o Workload, staffing, team dynamics, debriefing at work<br>o Curriculum design<br>o Safety/hygiene related to COVID-19<br>o Interdisciplinary workforce	"*the workplace environment…the culture that we're building in [the] workplace*" – Participant 17
	**Society & Culture**	▪ Alignment of societal expectations and clinician's role<br>▪ Discrimination and overt and unconscious bias	o Sociocultural expectations<br>o Sociocultural norms	"*Asian culture, people are expected to be tough*" – Participant 17
	**Rules & Regulations**	▪ Documentation and reporting requirements<br>▪ National and state policies and practices	o Policies related to personal, teaching, research requirements/equipment and the wider healthcare system	*"…the existing health system [has] some handicap…*" – Participant

		▪ Shifting systems of care and administrative requirements	o Good human resource system	15
**Individual Factors**	**Personal Factors**	▪ Personality traits<br>▪ Personal values, ethics, morals<br>▪ Level of engagement/connection to meaning & purpose in work<br>▪ Sense of meaning<br>▪ Work□ life integration<br>▪ Financial stressors/economic vitality<br>▪ Relationships and social support<br>▪ Physical, mental & spiritual well-being	o Personal/home/family life<br>o Sense of meaning/purpose/ responsibility<br>o Social support from families, friends, colleagues	"…*physical wellbeing…interpersonal relationship…personality traits…sense of self awareness…able to set boundaries*" and "*as human beings, we all need a sense of purpose, a sense of hope*" – Participant 1
	**Skills & Abilities**	▪ Clinical competency<br>▪ Coping skills<br>▪ Resilience skills/practice<br>▪ Mastering new technologies<br>▪ Teamwork skills	o Coping strategies<br>o Navigating new technologies due to COVID-19<br>o Professional competency<br>o Capacity to move on and overcome obstacles<br>o Self-imposed expectation management	"…*eh Netflix time? And shopping time definitely helps*"– Participant 3<br><br>"*you just need to learn to live with that and move on…*" – Participant 9

Abbreviation. NAM = National Academy of Medicine model.
